# Limited health literacy is associated with low glomerular filtration in the Chronic Renal Insufficiency Cohort (CRIC) study

**DOI:** 10.5414/CN108062

**Published:** 2013-11-13

**Authors:** Ana C. Ricardo, Wei Yang, Claudia M. Lora, Elisa J. Gordon, Clarissa J. Diamantidis, Virginia Ford, John W. Kusek, Amada Lopez, Eva Lustigova, Lisa Nessel, Sylvia E. Rosas, Susan Steigerwalt, Jacqueline Theurer, Xiaoming Zhang, Michael J. Fischer, James P. Lash

**Affiliations:** 1 Department of Medicine, University of Illinois at Chicago, Chicago, IL,; 2 Center for Clinical Epidemiology and Biostatistics, University of Pennsylvania, Philadelphia, PN,; 3 Feinberg School of Medicine, Northwestern University, Chicago, IL,; 4 University of Maryland School of Medicine, Baltimore, MD,; 5 National Institute of Diabetes and Digestive and Kidney Diseases, National Institutes of Health, Bethesda, MD,; 6 Tulane University, New Orleans, LA,; 7 Department of Medicine, University of Pennsylvania/Philadelphia VA Medical Center, Philadelphia, PN,; 8 St. John’s Health System, Detroit, MI,; 9 Division of Nephrology, Department of Medicine, MetroHealth Medical Center, Cleveland, OH, and; 10 Center for Management of Complex Chronic Care, Jesse Brown VA Medical Center, Chicago, IL, USA

**Keywords:** chronic kidney disease, health literacy

## Abstract

Background: Low health literacy in the general population is associated with increased risk of death and hospitalization. The evaluation of health literacy in individuals with predialysis chronic kidney disease (CKD) is limited. Methods: We conducted a cross-sectional study to evaluate the associations of limited health literacy with kidney function and cardiovascular disease (CVD) risk factors in 2,340 non-Hispanic (NH) Whites and Blacks aged 21 – 74 years with mild-to-moderate CKD. Limited health literacy was defined as a Short Test of Functional Health Literacy in Adults (STOFHLA) score ≤ 22. Outcomes evaluated included estimated glomerular filtration rate (eGFR), 24-hour urine protein excretion, and CVD risk factors. Results: The prevalence of limited health literacy was 28% in NH-Blacks and 5% in NH-Whites. Compared with participants with adequate health literacy, those with limited health literacy were more likely to have lower eGFR (34 vs. 42 mL/min/1.73 m^2^); higher urine protein/24-hours (0.31 vs. 0.15 g); and higher self-reported CVD (61 vs. 37%); and were less likely to have BP < 130/80 mmHg (51 vs. 58%); p ≤ 0.01 for each comparison. After adjustment, limited health literacy was associated with self-reported CVD (OR 1.51, 95% CI 1.13 – 2.03) and lower eGFR (β –2.47, p = 0.03). Conclusion: In this CKD cohort, limited health literacy was highly prevalent, especially among NH-Blacks, and it was associated with lower eGFR and a less favorable CVD risk factor profile. Further studies are needed to better understand these associations and inform the development of health literacy interventions among individuals with CKD.

## Introduction 

Health literacy is defined as “the degree to which individuals have the capacity to obtain, process and understand basic health information and services needed to make appropriate health decisions” [1]. The 2003 National Assessment of Adult Literacy estimated that 26% of adults in the U.S. had only basic or below basic health literacy skills [2] and therefore may have difficulty locating and understanding health-related information and services. In the general population, limited health literacy has been associated with adverse health outcomes including increased hospitalizations rates, use of emergency rooms, and delayed diagnosis [3, 4, 5, 6]. In addition, limited health literacy is associated with poor control of cardiovascular disease risk factors [7] and is an independent predictor of all-cause mortality in the general population [8], as well as in patients with end-stage renal disease [9, 10]. Moreover, the economic burden of limited health literacy is substantial, and is estimated to account for up to 5% of the total health care cost per year [11]. Therefore, the potential for health literacy interventions to improve health outcomes has been recognized and targeted as a high priority area of investigation [12]. 

The management of chronic kidney disease (CKD) generally requires lifestyle changes and adherence to complex medication and dietary regimens which may be particularly challenging for individuals with limited health literacy [13, 14]. Despite its potential health implications, the associations of limited health literacy with the health of individuals with mild-to-moderate chronic kidney disease are not well understood. Because of the known association of limited health literacy with poorer health outcomes and limited use of health care services [6], we hypothesized that limited health literacy would be significantly associated with lower levels of kidney function and higher prevalence of cardiovascular risk factors. We evaluated these associations in the Chronic Renal Insufficiency Cohort (CRIC) Study. 

## Subjects and methods 

### Study design and participants 

The CRIC Study was established in 2001 by the National Institute of Diabetes, Digestive, and Kidney Diseases (NIDDK) to improve our understanding of CKD and its relationship to cardiovascular disease. The design, methods, and the characteristics of the CRIC Study participants have been previously reported [15, 16]. In brief, the CRIC Study is an ongoing prospective cohort of adults with mild-to-moderate CKD (estimated glomerular filtration rate (eGFR) 20 – 70 mL/min/1.73 m^2^), recruited from seven U.S. clinical centers between 2003 and 2007. Sociodemographic characteristics, medical history and medications are self-reported; blood pressure (BP) and anthropometric measurements are obtained using standard methods at annual visits. 

An evaluation of health literacy using the Short Test of Functional Health Literacy in Adults (STOFHLA) was introduced into the CRIC Study protocol after 2008. We conducted a cross-sectional analysis of all non-Hispanic Black and White CRIC Study participants who were alive and actively enrolled in the study after the STOFHLA was introduced into the study protocol (n = 2,340 out of 2,565 eligible). The study protocol was approved by the Institutional Review Board of each participating institution and is in accordance with the principles of the Declaration of Helsinki. All participants provided written informed consent. 

### Exposure measure 

The primary independent variable of interest was health literacy as measured by the reading comprehension section of the STOFHLA [17], completed during a scheduled annual visit which occurred after a median of 4.7 years (interquartile range 3.9 – 5.1) after study enrollment. This abbreviated form of the STOFHLA assesses an individual’s ability to read and understand health-related prose and documents. This test includes two reading passages which use the Cloze procedure [18] (passages are missing every fifth to seventh word): the first selected from instructions written for patients receiving an upper gastrointestinal series (Gunning-Fog Index readability grade 4.3 [19]); and the second from the patient’s “Rights and Responsibilities” section of a Medicaid application form (Gunning-Fog Index readability grade 10.4). The STOFHLA is a 7-minute timed test that includes 36 multiple-choice items, worth 1 point each (total score ranges from 0 to 36), with higher scores indicating higher literacy levels. The test was completed in English and it was administered by trained personnel. Individuals who indicated that they could not read at all (n = 97) were assigned a score of 0. Limited health literacy was defined as inadequate/marginal health literacy (STOFHLA score from 0 to 22), and adequate health literacy was defined as a score from 23 to 36 [20, 21]. 

### Outcome measures 

Study outcomes included eGFR using the four-variable Modification of Diet in Renal Disease (MDRD) equation [22], cardiovascular risk factors (BP < 130/80 mmHg, low-density lipoprotein (LDL) cholesterol ≤ 100 mg/dL, and glycated hemoglobin (HbA1c) < 7%), and self-reported history of cardiovascular disease (defined as answering “yes” to having ever been diagnosed with at least one of the following: myocardial infarction, prior revascularization, heart failure, stroke or peripheral arterial disease). All outcomes measurements were obtained at the time of the STOFHLA administration, except LDL cholesterol level which was measured within 1 year of the STOFHLA. 

### Statistical methods 

Descriptive statistics were summarized as mean (SD) or median (interquartile range) for continuous variables, and frequency with proportions for categorical variables. Sociodemographic and clinical characteristics of individuals with inadequate/marginal health literacy were compared with those with adequate health literacy using t-tests or χ^2^-tests as appropriate. To evaluate the association between health literacy and each outcome of interest, we fitted linear regression models for continuous outcomes and logistic regression models for binary outcomes, and adjusted for pre-specified pertinent sociodemographic and clinical measures. We tested the interaction between health literacy and race/ethnicity by adding a product term of these two variables to each model. All hypothesis tests were 2-sided with α-level of 0.05. All statistical analyses were conducted using SAS, version 9.2 (SAS Institute, Cary, NC, USA). Due to the CRIC Study protocol design, not all participants had a serum LDL cholesterol level measured near the STOFHLA administration; therefore only 1675 individuals were included in the regression models where LDL was the outcome. Data were missing in < 5% of participants for the remaining outcomes (61 for eGFR, 20 for blood pressure, and 48 for HbA1c); therefore, these individuals were excluded from regression analyses. 

## Results 

### Characteristics of study participants 

The overall prevalence of limited (inadequate or marginal) health literacy, defined as a STOFHLA score ≤ 22, was 16% (381 out of 2,340), 28% in non-Hispanic Blacks and 5% in non-Hispanic Whites, p < 0.001. The median (interquartile range) STOFHLA score was 34 (5) for the overall cohort, 13 (18) for participants with limited health literacy, and 35 (3) for those with adequate health literacy. Compared with participants with adequate health literacy, those with limited health literacy were more likely to be older (66 vs. 62 years); have annual household income ≤ $20,000 (51% vs. 19%); and possess less than a high school education (44% vs. 7%); p < 0.001 for each comparison (Table 1). Participants with limited health literacy were also more likely to have lower mean eGFR (34 vs. 42 mL/min/1.73 m^2^), and were less likely to achieve BP < 130/80 mmHg (51 vs. 58%), compared with participants with adequate health literacy (p ≤ 0.01 for each comparison). Moreover, individuals with limited health literacy were more likely to self-report history of any cardiovascular disease (61 vs. 37%), which was due to higher reporting of myocardial infarction/prior revascularization (36 vs. 26%), congestive heart failure (23 vs. 10%), stroke (27 vs. 10%) and peripheral vascular disease (13 vs. 7%), p < 0.001 for each comparison (Table 1). We found no significant differences by gender, health insurance status, or use of angiotensin-converting enzyme inhibitors and angiotensin receptor blockers between individuals with low and adequate health literacy (p > 0.05). We conducted additional analyses to compare selected demographic and clinical characteristics among CRIC participants who were included (n = 1,675) vs. excluded (n = 665) from regression analyses of LDL cholesterol due to missing data. Compared with participants included, those who were excluded had lower eGFR (42 vs. 39 mL/min/1.73 m^2^ p = 0.002) and were more likely to be female (45 vs. 50%, p = 0.03). We found no significant differences in age, race/ethnicity distribution or educational attainment. 

**Figure 1.**
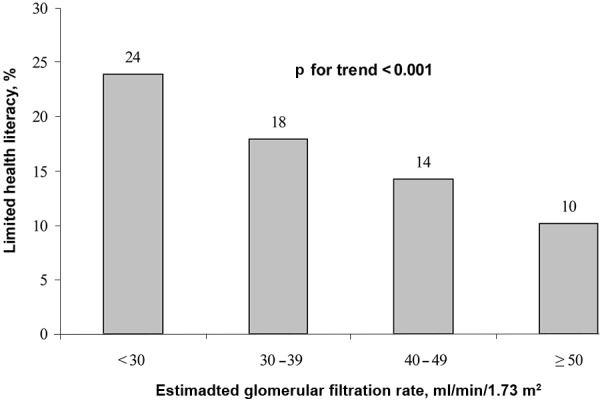
Prevalence of limited health literacy by estimated glomerular filtration rate.

In analyses stratified by eGFR, the prevalence of inadequate/marginal health literacy was highest (24%) among individuals with eGFR < 30 mL/min/1.73 m^2^, compared with 18%, 14% and 10% in participants with eGFR 30 – 39, 40 – 49 and ≥ 50, respectively; p < 0.001 (Figure 1). 

### Association between health literacy and selected outcomes 

In adjusted analyses, individuals with limited health literacy were more likely to self-report a history of cardiovascular disease compared with participants with adequate health literacy (OR 1.51, 95% CI, 1.13 – 2.03) (Table 2). On average, the mean eGFR was 2.47 mL/min/1.73 m^2^ lower in individuals with limited vs. adequate health literacy (p = 0.03) (Table 2). There was no significant independent association between health literacy and blood pressure, HbA1c or LDL cholesterol goals. We did not find a significant interaction between health literacy and race/ethnicity for any of the outcomes evaluated. 

## Discussion 

To our knowledge, this is the largest study examining health literacy in a diverse cohort of individuals with mild-to-moderate CKD. We found that 16% of participants had limited health literacy, and that these individuals were more often non-Hispanic black and had significantly lower educational attainment. Limited health literacy, as measured by the STOFHLA, had a strong and independent association with self-reported cardiovascular disease and low eGFR. 

The prevalence of limited health literacy in individuals with end-stage renal disease who are on maintenance dialysis has been found to be between 16% and 32% [9, 10, 23, 24, 25, 26]. However, studies assessing health literacy in predialysis CKD are scarce, with only one other published study reporting on the prevalence of limited health literacy in this population [26]. In this study, Wright et al. [26] used the Rapid Estimate of Adult Literacy in Medicine (REALM) to evaluate health literacy in 399 mainly white individuals recruited from a single nephrology clinic. Even though a different instrument was used, the 18% prevalence of limited health literacy reported in that study is comparable to the 16% observed in our study which had a much larger and diverse sample. 

We found that individuals with limited health literacy had significantly lower eGFR, which was significant even after accounting for educational attainment and other sociodemographic and clinical characteristics including blood pressure and diabetes. However, this was a cross-sectional study and therefore the directionality of this association cannot be established. Nonetheless, there are compelling reasons to believe that health literacy may be a predictor of kidney disease progression which include the high prevalence of medication non-adherence and lack of disease knowledge among patients with limited health literacy [14, 27, 28]. Future longitudinal studies are needed to address this question. 

Furthermore, we found a significant association between limited health literacy and self-reported history of cardiovascular disease. Although we could not evaluate the mediators of this association, health literacy might affect cardiovascular outcomes through a number of pathways including medication adherence, access to care, communication with health care providers, disease knowledge, and self-efficacy [13, 27, 29, 30, 31, 32, 33]. For instance, limited health literacy may lead to less engagement with care and poor management of chronic diseases that can lead to cardiovascular disease such as hypertension and diabetes. Moreover, in a recent cross-sectional study, Wright-Nunez et al. [34] quantified perceived kidney disease knowledge in 399 patients with predialysis CKD using a survey questionnaire. In adjusted analyses, they found that lower perceived knowledge was significantly associated with limited health literacy, and higher perceived knowledge was associated with higher odds of patient satisfaction with physician communication. These findings are important because they suggest that efforts to incorporate an assessment of health literacy into clinical practice and to modify patient-provider communication based on this assessment, may lead to improvements in disease understanding, patient satisfaction, and ultimately health outcomes. Moreover, future evaluation of literacy-sensitive self-management and disease knowledge interventions might also contribute to improve the health of patients with chronic kidney disease, as has been shown in patients with other chronic conditions such as chronic obstructive pulmonary disease [35] and heart failure [36]. 

Similar to other studies [9, 10, 23], we found that individuals with limited health literacy were more likely to be non-Hispanic black and have lower educational attainment. Even though health literacy and educational attainment are highly correlated [6, 8], more than half of our study participants with inadequate/marginal health literacy had at least a high school diploma. This finding suggests that educational attainment may not be a good indicator of a patient’s ability to navigate the health care system and to properly adhere to complex medication regimens [8]. Instead, assessment of functional health literacy might provide a more comprehensive evaluation of a patient’s understanding of medications, self-care, instructions and follow-up plans. Moreover, racial disparities have been previously observed in perceived knowledge about CKD and end-stage renal disease among patients cared for by nephrologists, with African Americans reporting having significantly less understanding than Asians or Caucasians [37]. The assessment of health literacy in the clinical setting might alert health care providers about the need for improving communication with patients and using appropriate materials for education and instructions. 

There are several limitations to this study. First, we conducted a cross-sectional analysis and therefore the directionality of the associations observed cannot be determined. Second, due to the CRIC Study visit schedule, not all participants had the health literacy assessment done concomitantly with the data collection for two of the outcomes evaluated. However, among adults, health literacy is considered to remain stable over time [38]. Third, although the STOFHLA is one of the best tools currently available to measure health literacy, it is limited by the fact that it only measures understanding of written information but does not account for other aspects of processing and understanding health-related information such as oral communication, ability to ask questions, and carry out basic math functions. Lastly, because the STOFHLA was introduced into the CRIC Study protocol several years after the study began, we were not able to evaluate health literacy in all CRIC participants; therefore, our findings might not be generalizable to all CRIC study participants or to the whole CKD population. 

In conclusion, limited health literacy was common in this large cohort of mild-to-moderate CKD. There was a significant association between limited health literacy and level of kidney function as measured by eGFR and self-reported cardiovascular disease. These findings suggest that it is important for clinicians to recognize that individuals with CKD and limited health literacy are a high risk group. Our findings suggest the need for longitudinal studies to evaluate health literacy as a predictor of clinical outcomes in patients with CKD, as well as the impact of health literacy interventions on their overall health. 

## Acknowledgments 

We acknowledge and are thankful for the time and commitment of the CRIC participants to the study. 

### Other sources of support that require acknowledgment 

Ana C. Ricardo: National Institutes of Health, National Institute of Diabetes and Digestive and Kidney Diseases Research Supplement to Promote Diversity in Health-Related Research to the Chronic Renal Insufficiency Cohort (CRIC) Study, U01 DK060980-04S2 (James P. Lash). 

Claudia M. Lora: Research support: University of Illinois at Chicago (UIC) Center for Clinical and Translational Science (CCTS) KL2 Scholars Program, Award Number UL1RR029879 from the National Center for Research Resources. 

Sylvia E. Rosas: Relevant support from the National Institute of Digestive, Diabetes and Kidney Diseases (R01 DK080033). 

Susan Steigerwalt: Relevant support from NIDDK for CRIC study. 

James P. Lash: Relevant support from the National Institute of Digestive, Diabetes and Kidney Diseases (R01 DK072231, U01 DK060980). 

## Funding source 

This work was funded under a cooperative agreement from the National Institute of Diabetes and Digestive and Kidney Diseases (NIDDK) (5U01DK060990, 5U01DK060984, 5U01DK06102, 5U01DK​0​61021, 5U01DK061028, 5U01DK60980, 5U01DK060963, 5U01DK060902) and an R01 DK072231 (HCRIC). In addition, this work was supported in part by institutional Clinical Translational Science Awards (CTSA) and other National Institutes of Health (NIH) grants: Johns Hopkins University UL1 RR-025005; University of Maryland GRCR M01 RR-16500; Case Western Reserve University Clinical and Translational Science Collaborative (University Hospitals of Cleveland, Cleveland Clinic Foundation, and MetroHealth) UL1 RR-024989; University of Michigan GCRC M01 RR-000042, CTSA UL1 RR-024986; University of Illinois at Chicago Clinical Research Center, M01 RR-013987-06; Tulane/LSU/Charity Hospital General Clinical Research Center RR-05096; University of Pennsylvania CTSA UL1 RR-024134; Kaiser NIH/NCRR UCSF-CTSI UL1 RR-024131, 5K24DK002651. Additional support was provided by the National Center for Minority Health and Health Disparities, NIH, and Department of Veterans Affairs Health Services Research and Development Service (MJF – Career Development Award). 

**Table 1.** Clinical and Demographic Characteristics by Health Literacy, n = 2,340.

Variables*		Health literacy		p
		Limited	Adequate	
		n = 381 (16%)	n = 1,959 (84%)	
Age, years, mean (SD)		66 (9)	62 (11)	< 0.001
Gender	Male	221 (58)	1,041 (53)	0.08
Income ≤ $20,000		193 (51)	365 (19)	< 0.001
Education	≤ 6th grade	14 (4)	2 (0.1)	< 0.001
	7 – 12 grade	154 (40)	136 (7)	
	≥ High school	213 (56)	1,821 (93)	
Health Insurance		359 (95)	1,865 (95)	0.41
Race	NH-White	67 (18)	1,155 (59)	< 0.001
	NH-Black	314 (82)	804 (41)	
Estimated GFR (mL/min/1.73 m^2^), mean (SD)		34 (19)	42 (18)	< 0.001
Urine protein, g/24 hour, median (IQR)		0.31 (0.10 – 1.17)	0.15 (0.07 – 0.59)	< 0.001
Hypertension		376 (99)	1779 (91)	< 0.001
Blood pressure < 130/80		191 (51)	1127 (58)	0.009
Diabetes		257 (68)	879 (45)	< 0.001
Glycated hemoglobin < 7% (Diabetics only)		142 (58)	508 (60)	0.52
LDL Cholesterol ≤ 100 mg/dL		155 (57)	742 (53)	0.19
Current Smoker		52 (14)	196 (10)	0.03
Total MET^†^, mean (SD)		145 (131)	202 (147)	< 0.001
Body mass index, kg/m^2^, mean (SD)		32 (8)	32 (8)	0.96
Family history of premature coronary heart disease		61 (16)	334 (17)	0.62
Medication use	ACE inhibitor/ARB	238 (63)	1283 (66)	0.31
	Statin	255 (68)	1201 (62)	0.03
	Aspirin	216 (57)	969 (50)	0.007
	Insulin	140 (37)	427 (22)	< 0.001
Perceived health	Good – excellent	156 (42)	1,300 (67)	< 0.001
	Fair	174 (47)	531 (27)	
	Poor	42 (11)	108 (6)	
Ever seen a nephrologist		348 (91)	1,627 (83)	< 0.001
Self-reported history of cardiovascular disease		231 (61)	728 (37)	< 0.001
Myocardial infarction/prior revascularization		138 (36)	518 (26)	< 0.001
Congestive heart failure		88 (23)	196 (10)	< 0.001
Stroke		103 (27)	197 (10)	
Peripheral vascular disease		51 (13)	142 (7)	< 0.001

GFR = glomerular filtration rate; IQR = interquartile range. *Values are presented as n (%) unless otherwise specified.^ †^Metabolic equivalent for all physical activity in the prior month.

**Table 2.** Logistic regression models comparing outcomes for limited vs. adequate health literacy.

Outcome	Unadjusted		Model 1*		Model 2^†^	
	β coefficient (SE) or OR (95% CI)	p	β coefficient (SE) or OR (95% CI)	p	β coefficient (SE) or OR (95% CI)	p
Estimated GFR	–8.03 (1.02)	< 0.001	–4.18 (1.16)	< 0.001	–2.47 (1.14)	0.03
Self-reported CVD	2.6 (2.08 – 3.26)	< 0.001	1.74 (1.32 – 2.3)	< 0.001	1.51 (1.13 – 2.03)	0.006
BP < 130/80 mmHg	0.74 (0.6 – 0.93)	0.009	0.97 (0.74 – 1.28)	0.82	0.97 (0.73 – 1.29)	0.86
Glycated hemoglobin < 7%^‡^	0.91 (0.68 – 1.21)	0.52	0.73 (0.51 – 1.04)	0.08	0.82 (0.56 – 1.21)	0.32
LDL cholesterol ≤ 100 mg/dL	1.19 (0.92 – 1.55)	0.19	1.00 (0.73 – 1.38)	0.97	0.9 (0.65 – 1.26)	0.55

*Adjusted for age, gender, race/ethnicity, clinical center, education, current smoking and BMI. ^†^In addition to variables included in Model 1, each model was adjusted for: eGFR: Prior contact with a nephrologist, systolic BP, diabetes, glycated hemoglobin, and use of ACEi/ARB. Self-reported cardiovascular disease: eGFR, diabetes, hyperlipidemia, systolic BP, use of ACEi/ARB and aspirin. BP < 130/80: eGFR, diabetes and number of BP-lowering medications. LDL cholesterol: diabetes, physical activity, and statin use. Glycated hemoglobin: Physical activity, insulin and prednisone use. ^‡^Only individuals with diabetes were included in these models (n = 1,088). ACEi = Angiotensin-converting enzyme inhibitor; ARB = Angiotensin receptor blocker; BP = blood pressure; BMI = body mass index; CVD = Cardiovascular disease; LDL = low density lipoprotein cholesterol; GFR = glomerular filtration rate.
